# Epithelial topography for repetitive tooth formation

**DOI:** 10.1242/bio.013672

**Published:** 2015-11-04

**Authors:** Marcia Gaete, Juan Manuel Fons, Elena Mădălina Popa, Lemonia Chatzeli, Abigail S. Tucker

**Affiliations:** 1 Department of Craniofacial Development and Stem Cell Biology, King's College London, London SE1 9RT, UK; 2 Department of Anatomy, Faculty of Medicine, Pontificia Universidad Católica de Chile, Santiago 8331150, Chile

**Keywords:** Successional tooth development, Sox2, Sox9, Odontogenesis, Organogenesis

## Abstract

During the formation of repetitive ectodermally derived organs such as mammary glands, lateral line and teeth, the tissue primordium iteratively initiates new structures. In the case of successional molar development, new teeth appear sequentially in the posterior region of the jaw from *Sox2^+^* cells in association with the posterior aspect of a pre-existing tooth. The sequence of molar development is well known, however, the epithelial topography involved in the formation of a new tooth is unclear. Here, we have examined the morphology of the molar dental epithelium and its development at different stages in the mouse *in vivo* and in molar explants. Using regional lineage tracing we show that within the posterior tail of the first molar the primordium for the second and third molar are organized in a row, with the tail remaining in connection with the surface, where a furrow is observed. The morphology and *Sox2* expression of the tail retains characteristics reminiscent of the earlier stages of tooth development, such that position along the A-P axes of the tail correlates with different temporal stages. *Sox9*, a stem/progenitor cell marker in other organs, is expressed mainly in the suprabasal epithelium complementary with *Sox2* expression. This *Sox2* and *Sox9* expressing molar tail contains actively proliferating cells with mitosis following an apico-basal direction. *Snail2*, a transcription factor implicated in cell migration, is expressed at high levels in the tip of the molar tail while E-cadherin and laminin are decreased. In conclusion, our studies propose a model in which the epithelium of the molar tail can grow by posterior movement of epithelial cells followed by infolding and stratification involving a population of Sox2^+^/Sox9^+^ cells.

## Abstract (precis)

**Summary:** This study proposes a model for repetitive tooth formation in which the epithelium can grow by the movement of epithelial cells followed by infolding and stratification of Sox2/Sox9 positive cells.

## INTRODUCTION

Teeth are important organs required for eating and speaking, and tooth loss is a common problem for society. The biological replacement of missing teeth is the ultimate goal for clinicians, either by implanting a bioengineered tooth or by stimulating dental tissue to form a new organ. To accomplish this goal it is necessary to understand the mechanisms involved during tooth initiation. Teeth can be formed *de-novo*, i.e. initiate for the first time from new placodes, or can be formed in succession, i.e. initiate from pre-existing dental tissue. This last method, known as successional tooth development, can be observed during the formation of molar teeth in mammals, including human permanent molars and mouse molar development. In both species, a single molar placode at the back of each jaw quadrant gives rise to three molars that sequentially bud off from each other (Lumsden, 1979; Ooe, 1979). In the case of the mouse, the first molar initially forms at embryonic day (E)11 as a thickening of the dental epithelium. The second molar subsequently develops from the posterior edge of the first molar at E16 (Kavanagh et al., 2007). Finally, the third molar forms postnatally from the distal edge of the second molar (Chlastakova et al., 2011). Using 3D reconstructions, the molars appear to grow as swellings on a mound of dental epithelium (Peterkova et al., 2014). Textbooks indicate that tooth initiation of the first teeth is different from the initiation of successional molars, and that a backward extension of the dental lamina burrows beneath the epithelium of the oral mucosa, successively giving rise to epithelial outgrowths forming the tooth germs for the second and third molars (Nanci, 2008). When the molar placode is dissected out and cultured *in vitro* at E12 the complete molar dentition forms in sequence maintaining the normal crown shapes and proportions (Lumsden, 1979). At this early stage of dental development, therefore, all the patterning information to create successional teeth is already present in the molar placode. Culturing the molar placode is a common tool in dental research and has been used to understand the control of the tooth number and cusp pattern (Jernvall and Thesleff, 2012; Kavanagh et al., 2007).

At E14.5, when the first molar tooth germ has reached the cap stage and the second molar is about to initiate, the dental epithelium extends out further than the tooth germ in the anterior and posterior direction. The ontogeny of the anterior region is well studied as this region contains rudimentary diastemal buds, of which the most posterior contributes to the first molar (Prochazka et al., 2010). The posterior region, which we refer to as the molar tail, is the site where new molars are added. The tail is distinguishable at E14.5 as a finger-shaped bulge at the most distal end of the first molar, corresponding to the future second molar tooth germ (Peterkova et al., 2014). This bulge is also observed at E17.5 and postnatal day (P)0, corresponding to the developing third molar (Chlastakova et al., 2011; Peterkova et al., 2014). It is not clear from what part of the dental epithelium, the second and third molars develop. Similarly in humans, the origin of the second permanent molar is controversial. It has been suggested that it could derive from a lingual epithelial projection of the first permanent molar, however, because the lingual projection of the first permanent molar is still discernible even in specimens with the second permanent molar, it is possible that the primordium is derived from the distal end of the dental lamina (Ooe, 1979). It is also not fully understood how the dental cells are arranged in the dental epithelium, for example, whether cells for the first, second and third molar are determined in rows at early stages and then expand distally, or whether they emerge after the predecessor tooth germ has started to develop.

The transcription factor SRY (sex determining region Y)-box 2 (Sox2) has been identified as a dental epithelial stem cell marker during mouse tooth development (Juuri et al., 2012). During successional tooth development, Sox2 is expressed in the dental lamina of primary molars and in the posterior tail (Juuri et al., 2013a). Moreover, Sox2^+^ cells have been shown to be involved in the control of the formation of successional teeth in animals with multiple generations of teeth (Gaete and Tucker, 2013; Juuri et al., 2013b). Tracking of Sox2^+^ cells has shown that they contribute to the epithelium of the developing second and third molars in the mouse (Juuri et al., 2013b). The key role of Sox2 in tooth development has been shown by conditionally deleting Sox2, resulting in aberrant formation of the dental epithelium (Juuri et al., 2013b). A second Sox transcription factor expressed during dental development is Sox9. Sox9 is a neural, pancreatic and pituitary stem/progenitor cell marker (Furuyama et al., 2011; Rizzoti et al., 2013; Scott et al., 2010). Although Sox9 is well studied in these organs its expression has only briefly been studied in the tooth, where it has been shown to be expressed in the dental epithelium including the stellate reticulum, the core of the epithelial organ of the tooth germ (Mitsiadis et al., 1998).

Here, we have studied the composition, the cellular origin and the fate of the molar tail. For this we have utilised immunofluorescence, transgenic reporter mice and 3D reconstructions to comprehensively examine the topography over this region. We observed that the molar tail contains different epithelial layers, similar to the enamel organ, and that its lingual basal epithelium and the adjacent oral epithelium strongly express Sox2, while the suprabasal dental epithelium strongly expresses Sox9. Lineage tracing to investigate the origin and fate of the tail of the molar showed that at E14.5 the tail contains cells destined to be part of the second and third molar. Additionally, clonal analysis in the mT/mG transgenic mice and the expression pattern of *Snail2* suggest the molar tail is formed by posterior cell movement, followed by an infolding process and lateral growth by stratification.

## RESULTS

### The dissected molar region gives rise to three molars in culture that appear sequentially from its posterior end

**Fig. 1.**
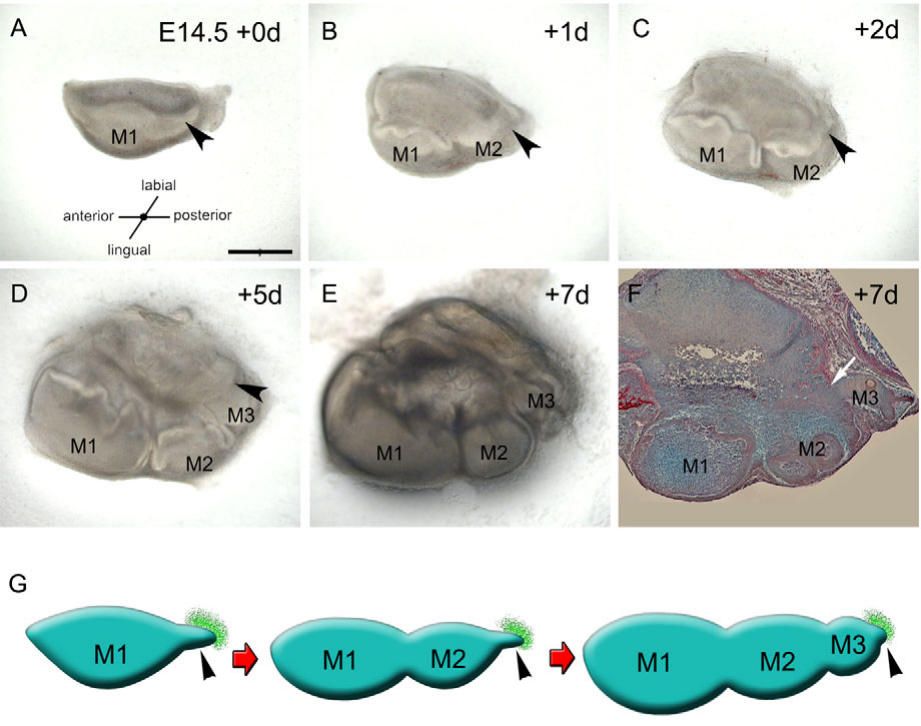
**Three molars successionally develop in culture.** (A-E) Development of a molar explant including the first molar germ and its tail after (A) 0 day, (B) 1 day, (C) 2 days, (D) 5 days and (E) 7 days. (F) Histological section of the explant in A-E after 7-days of culture, arrow shows the continuity between the dental and oral epithelium. Arrowheads show the distal molar tail. (G) Schematic representation of the development of the three molars. Oral epithelium is shown in green and tooth germs in blue. Arrow shows continuity of tooth germ epithelium with suprajacent epithelium. M1, M2 and M3 are the first, second and third molar respectively. Scale bar in A: 500 µm, B-E similar magnification for A.

In order to visualise morphological changes and the timing of appearance of successional molars in culture, we dissected out the first molar placode and its posterior tail (Fig. 1A, M1 and arrowhead) and performed organotypic culture over 7 days. At 1 day of culture, the second molar germ started to develop posterior to the first molar from the region of the tail (Fig. 1B, M2) and continued growing at 2 days (Fig. 1C, M2). From day 5 to 7, the third molar germ appeared and grew posterior to the second molar (Fig. 1D,E, M3). All these molars germs grew sequentially in relation to a suprajacent oral epithelium (Fig. 1G, green sheet in schematic) that appeared in continuity with the tip of the molar tail in culture (Fig. 1B-D, arrowhead; Fig. 1F, arrow). To get an idea of the dynamic movements involved we constructed a morphed movie from the sequential images (Movie 1).

### Cells at the tip of the molar tail do not contribute to the formation of the immediate successional molar

**Fig. 2.**
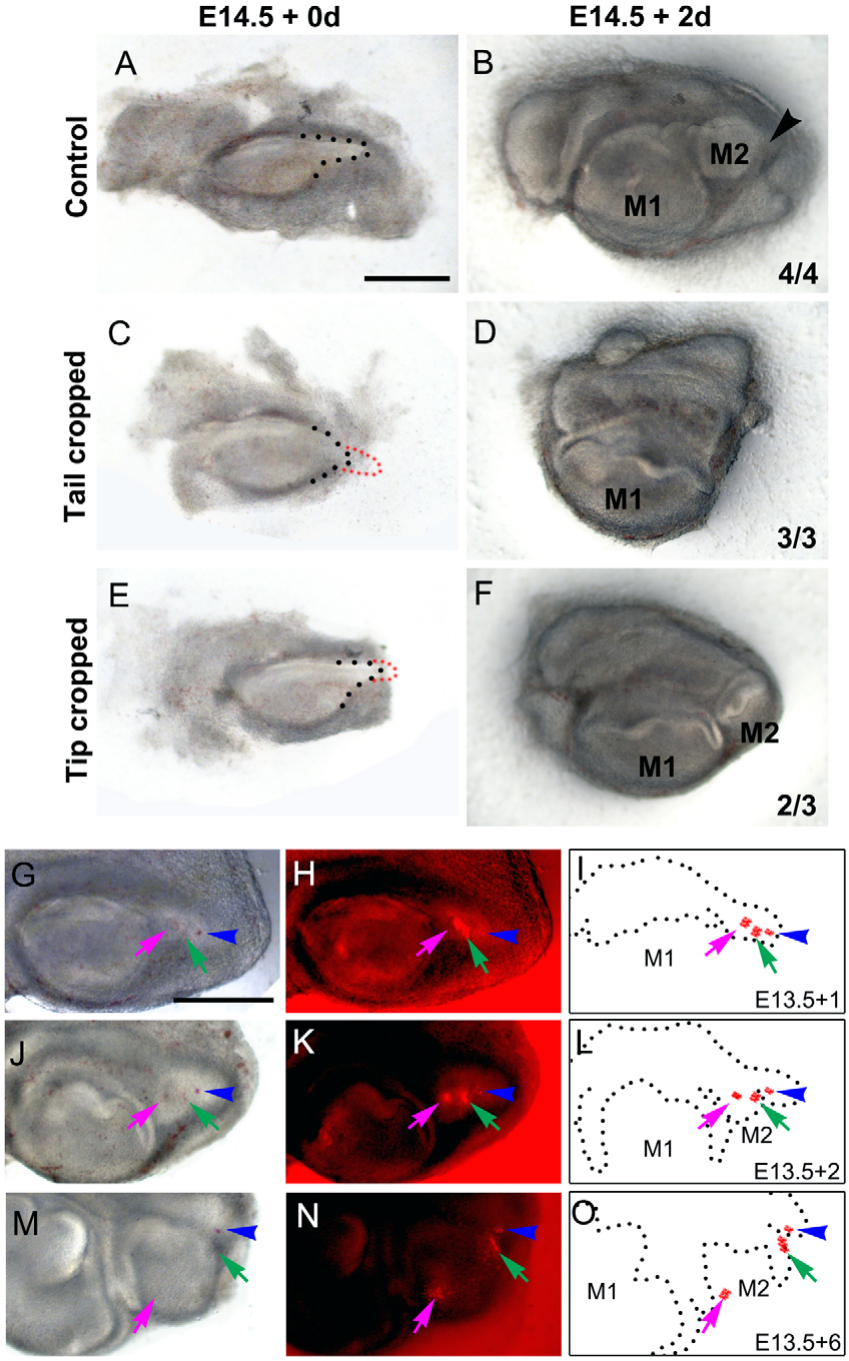
**The successional molars are generated from the molar tail.** (A-F) Molar cultures from E14.5 after (A,C,E) 0 day and (B,D,F) 2 days. (A,B) Control cultures, (C-F) molar tail ablations. The complete (C,D) or only the tip (E,F) of the molar tail were dissected. The molar tail and cropped region are outlined with black and red dots respectively. (G-O) E13.5 molar tail labelled with DiI and followed in culture after 6 days. (G-I) 1 day, (J-L) 2 days and (M-O) 6 days of culture. (G-I) DiI labelling was performed at the anterior (magenta arrows), medial (green arrows) and posterior (blue arrowheads) regions of molar tail after 1 day of culture. Arrowhead indicates the developing third molar field. (J-O) DiI labels were incorporated in the developing second (magenta and green arrows) and third (blue arrowheads) molars. (G,J,M) Brightfield images, (H,K,N) eepifluorescence images, (I,L,O) schematic diagrams. M1, M2 and M3 are the first, second and third molar respectively. Scale bars: 500 µm.

The fact that new molars appear in the region of the molar tail, prompted us to identify what portion of the tail contributes to the second and third molars. To assess this, we performed molar explants and manually cropped the molar tail at the tip (cutting out one third of the tail; Fig. 2C) or close to the base (cutting out two thirds of the tail; Fig. 2E). When the tail was removed at the base the formation of the second molar was inhibited in 3 of 3 cases (Fig. 2C,D). However, when only the tail tip was cropped the second molar germ was formed in 2 of 3 cases, but no extra budding toward a third molar formation was observed compared to control cultures (Fig. 2F compare to arrowhead in Fig. 2B).

We analysed if at E14.5 the first molar tail contained predetermined regions for the second and third molars by lineage tracing the tail (*n*=4). We labelled the tail of the E14.5 first molar using DiI in three regions along the tail and followed molar development in culture. None of the DiI spots were diluted or visibly expanded. Some DiI spots appeared to project into the dental mesenchyme region due to epithelial cells wrapped around the mesenchyme as the tooth developed. From anterior to posterior, the first and the second DiI labels, were incorporated into the developing second molar (Fig. 2G-O, green arrows). The posterior label, which slightly touched the second label at day 0, was maintained at the tip, which started to acquire third molar morphology after 6-day of culture (Fig. 2G-O, blue arrows). These results indicate that at E14.5 the cells in the first molar tail have two different fates: the anterior region contribute to the second molar and the most posterior tip to the third molar.

### The posterior tail of the molar is continuous with the superficial epithelium

**Fig. 3.**
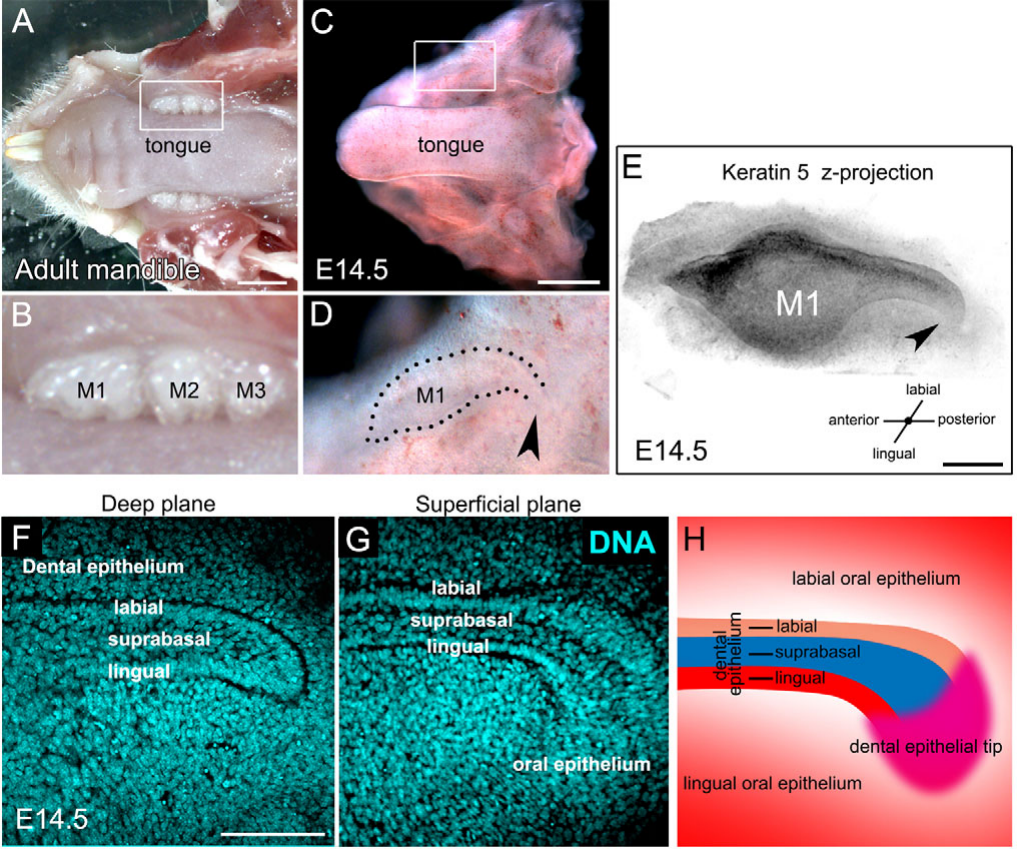
**The molar tail is linked to the superficial epithelium.** (A) Adult and (C) E14.5 dissected mandible. (B,D) Enlarged view of the framed regions in A and C, respectively. (E) *Z*-stack projection of a molar dental epithelium immunostained for Keratin 5. Arrowheads in D and E show the connection between the molar tail and the superficial epithelium. (F,G) DNA staining of an E14.5 molar tail showing a (F) deep and (G) superficial plane. (H) Schematic of the molar tail showing the oral and dental epithelium. Scale bars: 2 mm in A; 1 mm in C; 200 µm in E; 100 µm in G.

Adult mice display three molars that develop sequentially toward the back of the mouth (Fig. 3A,B). As shown in Fig. 1, new molars appear from the posterior tail of the molar dental epithelium, we therefore aimed to analyse the morphology of this region in whole mount dissection of non-cultured samples. From an oral view of a freshly dissected E14.5 mandible, the entire molar dental epithelium acquires a dolphin-like shape: with an anterior mouth; a middle belly, which corresponds to the first molar germ; and a posterior tail that forms a curve toward the oro-lingual plane (Fig. 3C,D). 3D reconstruction of confocal optical sections of Keratin 5 labelled epithelium, showed that the tip was continuous with the oral surface and therefore the posterior border was not clearly defined (Fig. 3D,E, arrowheads). Using nuclear staining, the tail could be subdivided into three distinct epithelial compartments: the basal layer, divided into lingual and labial dental epithelium, and a suprabasal epithelium flanked by the two basal layers (Fig. 3F,G, scheme in Fig. 3H). The epithelium of the tail therefore has diverse zones with its most distal tip connected to the superficial epithelium. The distal end of the tail will be referred to as the dental epithelial tip (Fig. 3G, scheme in Fig. 3H).

### No evidence of localised proliferation but orientated cell division in the molar tail

**Fig. 4.**
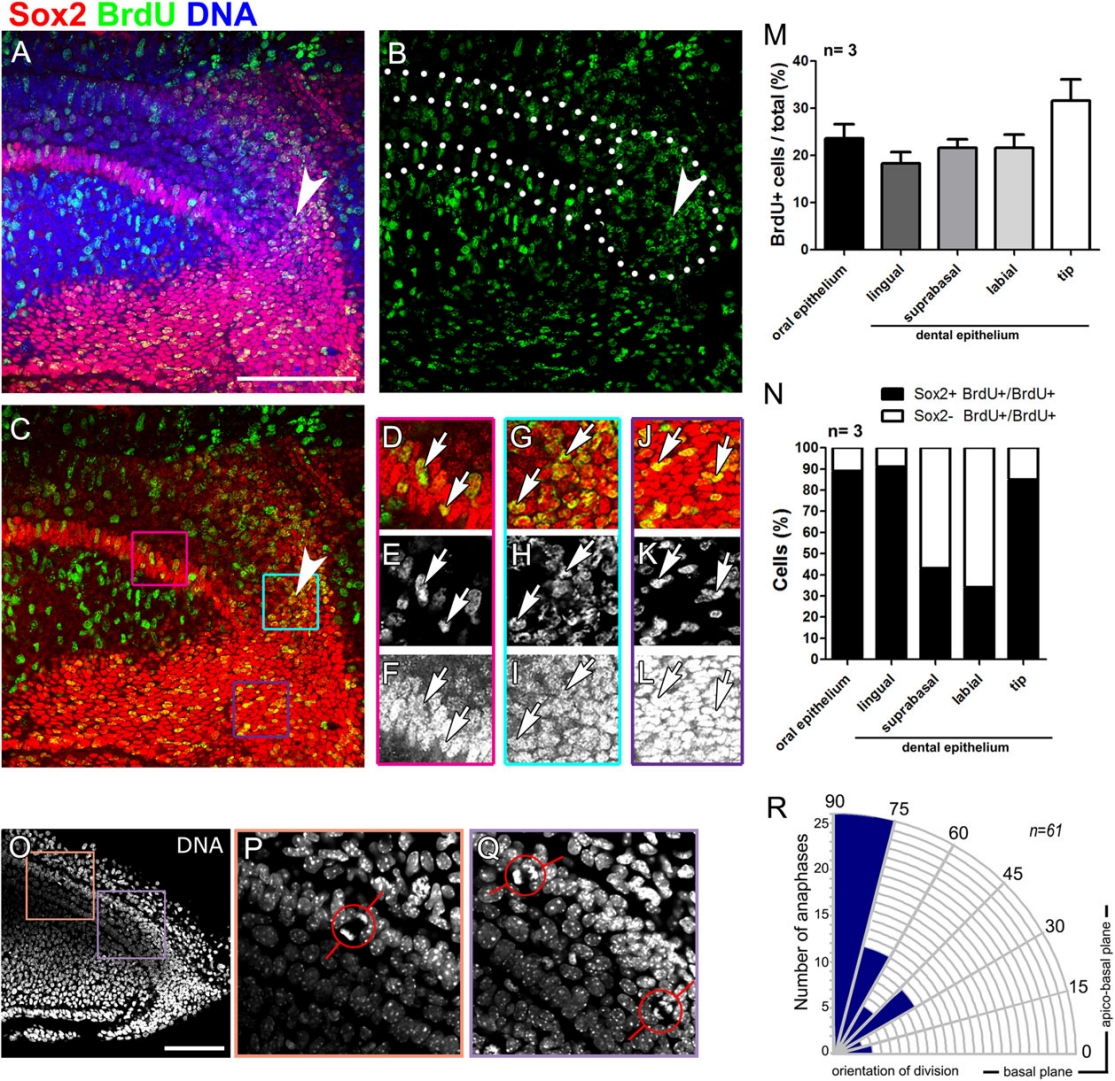
**Proliferation at the molar tail is homogenous and oriented in the bucco-lingual plane.** (A-L) Immunofluorescence of E14.5 molar tail stained for BrdU (green), Sox2 (red) and DNA (blue). BrdU injection was performed at E13.5 and fixed at E14.5. Arrowheads in A-C indicate the dental epithelial tip. Arrows in D-L indicate Sox2+ cells that colocalize with BrdU. (D,G,J) Sox2 and BrdU channels, (E,H,K) BrdU channel, (F,I,L) Sox2 channel. D-F, G-I and J-L are magnifications of the lingual dental epithelium, epithelial tip and oral epithelium, respectively in the region indicated by the color code in C. (M,N) Quantification of BrdU-positive cells (M) and of Sox2+/BrdU+ and Sox2−/BrdU+ cells (N) in the epithelial layers of the molar tail. Bars and error bars in M correspond to mean±s.e.m. (O-Q) DNA staining of the tail of the first molar. (P,Q) Magnifications of the framed areas in O. Anaphases are highlighted by the red circles, with lines indicating the orientation of division. (R) Plot of the anaphase axis orientation, an axis parallel to basal lamina is 0° and a perpendicular axis is 90°. The number of anaphases detected are shown in blue. Scale bars: 100 µm.

To investigate whether localised proliferation of the epithelial tip was involved in successional tooth development we assessed the levels of proliferation by BrdU labelling. Growth and elongation of the molar tail was prominent between E13.5 to E14.5, and so this time point was chosen for analysis. Mouse embryos were exposed to BrdU at E13.5 for 24 h and fixed at E14.5. BrdU incorporation was quantified in the oral epithelium, lingual, suprabasal, and labial dental epithelium and in the dental epithelial tip (Fig. 4A-N). The dental epithelial tip incorporated BrdU in 31% of the cells (Fig. 4A-C, arrowhead; Fig. 4G-I,M, zoom) with a slightly reduced incorporation in the rest of the tail (19% to 23%; Fig. 4D-F,J-L,M), although the difference was not statistically significant (Fig. 4M). Interestingly, the nuclei in the tip had punctate BrdU retention, compared to the rest of the tissue, which showed a mix of punctate and homogeneous BrdU labelling (Fig. 4H compare to Fig. 4E,K). Punctate BrdU retention could indicate greater dilution of the initial incorporation due to replication. In addition to proliferation, changes in the orientation of cell division have been shown to lead to changes in the shape of developing organs (Economou et al., 2013; Panousopoulou and Green, 2014). We therefore assessed the orientation of mitotic figures in anaphase in the molar tail, focusing on couples of segregated chromosomes located in the basal-suprabasal epithelium (Fig. 4O-R). Interestingly, the majority of the mitotic figures followed an apico-basal plane (Fig. 4R). Cell division is therefore not random but orientated in the molar tail.

### Evidence of cell migration and infolding in the molar tail

**Fig. 5.**
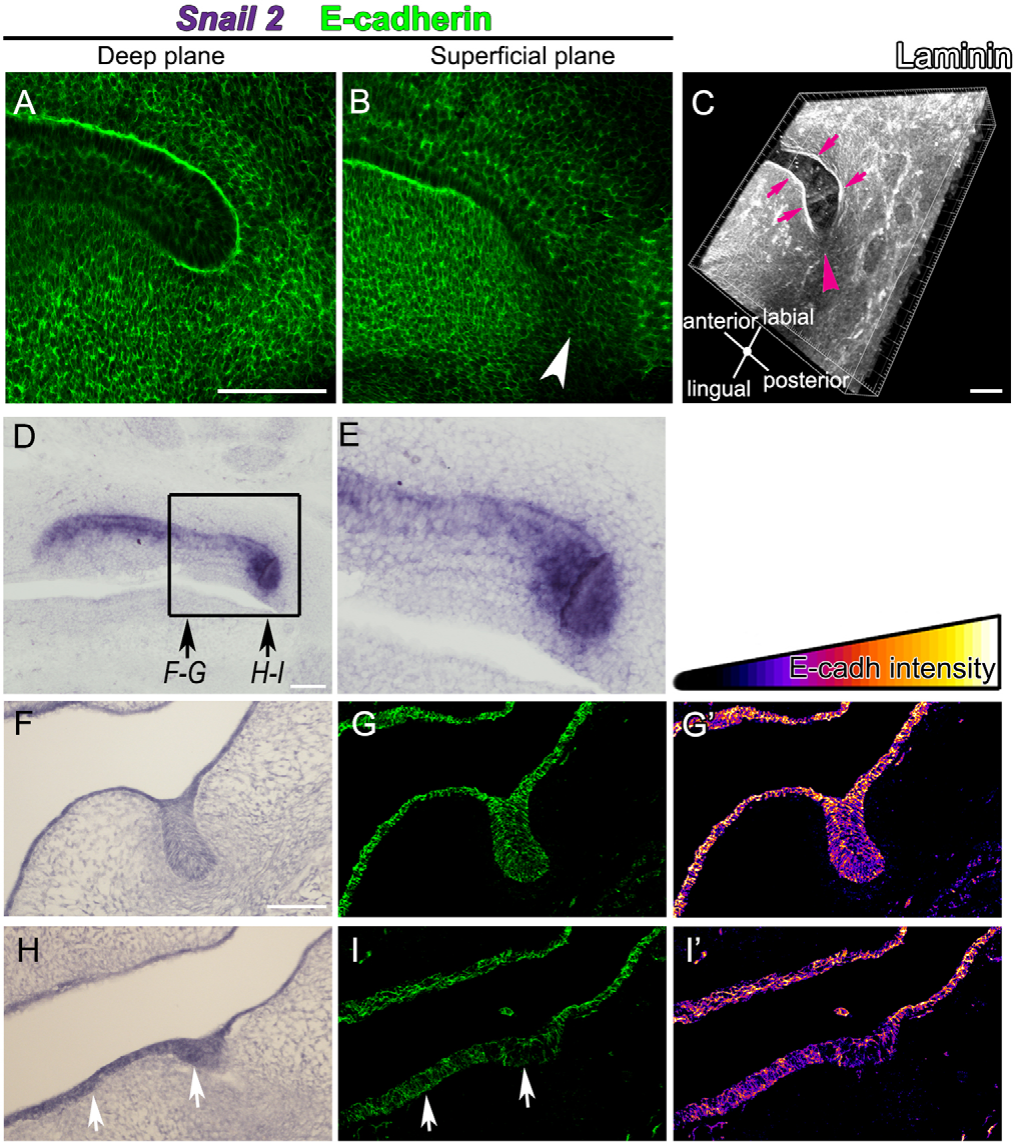
**High levels of Slug2 are associated with the tail tip.** (A,B) Single confocal sections of the molar tail stained for E-cadherin (green), showing deep (A) and superficial (B) planes. Note the reduction in intensity of E-cadherin superficially at the tip of the molar tail (arrowhead). (C) *Z*-projection of the molar tail stained for Laminin (white). Arrows show strong expression the edge of the molar tail and arrowhead shows decreased expression in the distal tip. (D-F,H) Snail2 *in situ* hybridization and (G-I) E-cadherin immunofluorescence in the molar developing region at E14.5. (D) Sagital section. The posterior tip is magnified in E as indicated. (F,H) Frontal sections in the levels indicated in D. Snail2 is detected in the epithelial tip of the tail and in the bud, where E-cadherin is decreased (see arrows in H,I). (G′,I′) Pseudocolored intensity of G,I. Scale bars: 100 µm.

The mechanism that drives the growth of the molar tail is unclear. Classical texts describe that new molars form as a posterior projection of the first molar dental epithelium. Our results show that the tail is also proliferating in a lingual-buccal direction, however this mechanism does not explain the observed posterior elongation of the molar tail. The transcription factor Snail is involved in the downregulation of E-cadherin to reduce intercellular adhesion required for epithelial cell movement (Thiery et al., 2009). Reduction of basement membrane proteins such as laminin are observed in Snail expressing cells (Haraguchi et al., 2008). In the molar tail, the deep and superficial epithelial layers expressed E-cadherin (Fig. 5A,B), however these levels decreased in the distal path close to the surface (Fig. 5B, arrowhead). Laminin consistently outlines the lingual and labial dental epithelia (Fig. 5C, arrows) but with reduced intensity at the tip (Fig. 5C, arrowhead). To confirm whether *Snail2* is expressed in this region, we performed *Snail2 in situ* hybridization at E14.5. We observed *Snail2* mRNA at high levels specifically in the molar tail in sagittal sections (Fig. 5D,E). Correlation between increase in *Snail2* levels and decrease in E-cadherin intensity were confirmed by *Snail2* and E-cadherin detection in sequential frontal sections (Fig. 5F-I, levels of sections indicated in Fig. 5E). For E-cadherin, pseudocolor was used to visualize changes in intensity (Fig. 5G′,I′). We observed that, matching the increase in *Snail2* mRNA, E-cadherin intensity was reduced in the tail tip (Fig. 5I, arrows; correlate to Fig. 5H) when compared to the dental epithelium of the forming first molar (Fig. 5G, correlate to Fig. 5F), which is at the cap stage at this time point.

**Fig. 6.**
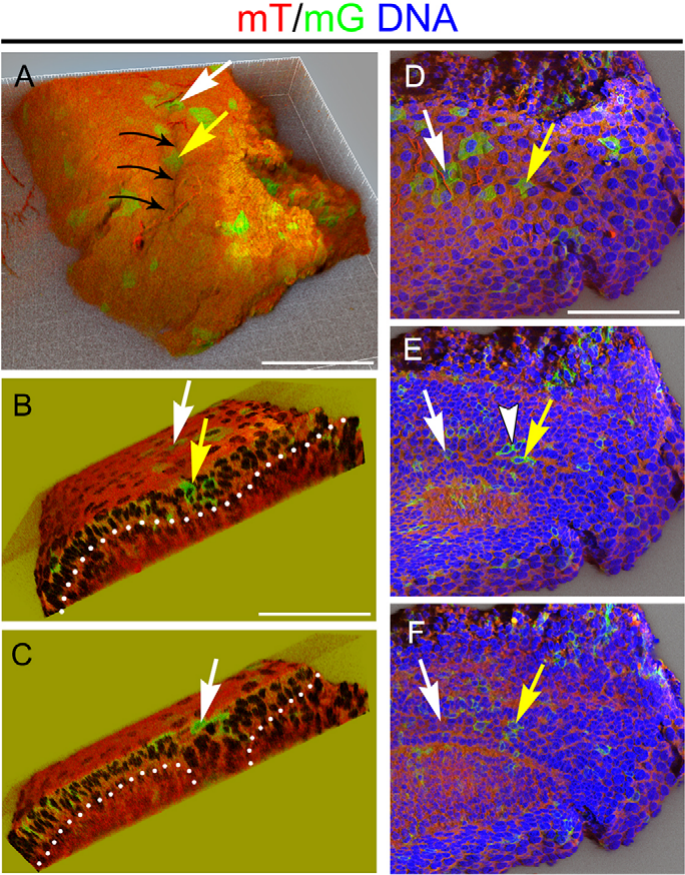
**Appearance of epithelial infolding in the molar tail.** Projections of clonal analysis in the molar tail using the mT/mG transgenic mice. Clones (mG) are in green, membrane (mT) in red and DNA in blue. Yellow arrows show posterior clones and white arrows show anterior clones in the molar tail. Arrowhead indicates suprabasal layer formation. Dotted lines show the basal lamina. (A) Black arrows highlight the dental furrow. (B) Posterior section with clones spanning the thickening. (C) Anterior section with superficial clones. (D-F) Superficial (D), medial (E) and deep (F) planes of the molar tail showing cell shape within the clones. Scale bars: 100 µm.

Movement of the cells could be coupled to changes in cell shape. In order to understand the cell shape and lineage of the cells of the tail of the molar placode we utilised double-fluorescent *R26R-mT/mG;R26R-CreER* transgenic embryos, which express a membrane-targeted Tomato (mT), a red fluorescent protein that can be excised after pharmacological induction of the Cre recombinase in a clonal manner. After recombination, cells express a membrane-targeted EGFP (mG) (Muzumdar et al., 2007). In the mT/mG mice induction was adjusted to allow labelling of one cell, hence every group of GFP expressing cells is likely to be a clonal group with the same origin. Similar approaches have been used for clonal analysis in the palate and hair (Economou et al., 2013; Sequeira and Nicolas, 2012). In agreement with our oriented cell division analysis and *Snail2* expression, the buccal dental epithelium contained groups of clonal cells that protruded toward the suprabasal dental epithelium (Fig. 6E, arrowhead). Most of the GFP-expressing groups of cells that were located superficially in the anterior region of the molar tail did not extend into the placode (Fig. 6A-F, white arrow). In contrast, groups of GFP-expressing cells in the tip of the tail extended from the surface to the basal layer (Fig. 6A,B,D-F, yellow arrow). Here, a furrow was observed in the surface of the epithelium (Fig. 6A, black arrows).

Collectively, the analysis of *Snail2*, E-cadherin and mT/mG expression suggest that the posterior end of the molar tail grows posteriorly, incorporating basal cells from the superficial epithelium by an infolding process. Then, a suprabasal layer is created by apico-basal proliferation and cell movement. Both mechanisms potentially provide a source of cells to create additional teeth.

### Sox2 and Sox9 are expressed in adjacent epithelial compartments within the molar tail

The re-iterative formation of new successional teeth requires stem cell activation to provide a constant source of cells. We therefore evaluated the expression of known epithelial stem and progenitor cell markers. Sox2 has recently been identified as a dental epithelial stem cell marker contributing to the formation all the epithelial lineages of the tooth (Juuri et al., 2012). By immunofluorescence, Sox2 expression was observed localised to the lingual dental epithelium and the tip of the tail at E14.5 (Fig. 4A-L). Sox2 was also expressed in the lingual oral epithelium that was coupled to the Sox2^+^ lingual dental epithelium (Fig. 4A,C). We investigated proliferation in the Sox2^+^ cells using our BrdU incorporation assay (injected at E13.5 fixed at E14.5). We observed that most of the BrdU^+^ cells of the lingual-oral, lingual-dental epithelium and dental epithelial tip were Sox2^+^ (85%-91%; Fig. 4N). In contrast, the proliferating cells that incorporated BrdU in the labial and suprabasal epithelium were predominately Sox2 negative (57%-66%; Fig. 4N). These results indicate that from the moment of the injection (E13.5) the proliferating cells were predominately Sox2^+^ cells.

**Fig. 7.**
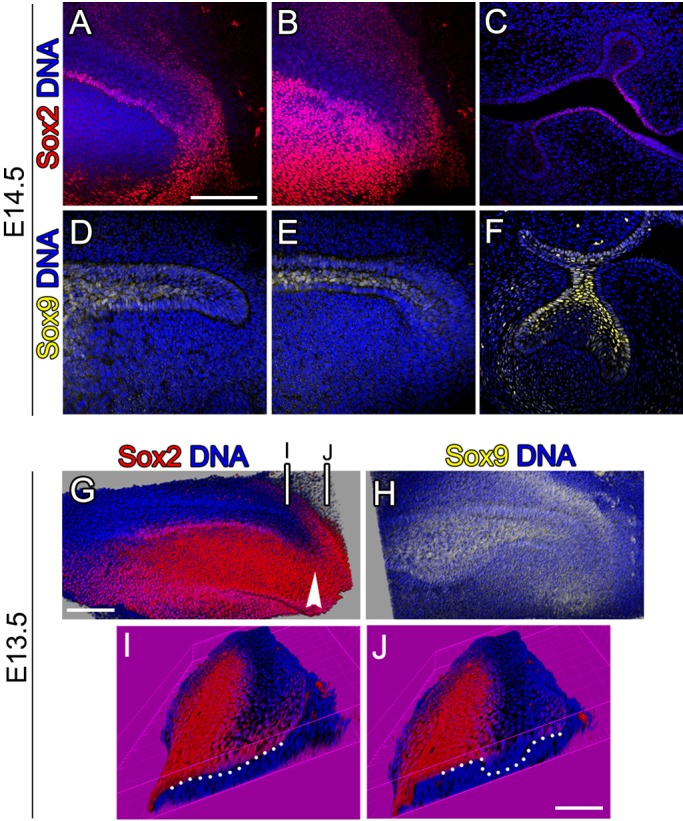
**Sox2 and Sox9 are expressed in the molar tail.** Immunofluorescence of the molar tail showing Sox9 (yellow) Sox2 (red) and DNA (blue) at E14.5 (A-F) and E13.5 (G-J). (A,D) middle and (B,E) superficial sections of the molar tail following superficial plane. (C,F) Frontal section of the first molar germ. (G-J) Projections of *Z*-stacks of the molar. Arrowhead shows Sox2^+^ cells behind the molar dental epithelium. (I,J) Posterior views of the planes marked in G. Dotted lines mark the basal lamina. Scale bars: 100 µm.

We also investigated the expression of Sox9, a second member of the family of Sox transcription factors known for its role as a progenitor cell marker (Furuyama et al., 2011; Rizzoti et al., 2013). Sox9, was expressed mainly in the suprabasal dental epithelium and in some cells of the labial and lingual dental epithelium (Fig. 7D-E). In frontal sections at the level of the first molar, Sox9 was expressed in the stellate reticulum, in the outer enamel epithelium and in the dental stalk (Fig. 7F). Interestingly, Sox9^+^ cells were few or absent in the Sox2^+^ regions: the oro-lingual epithelium and the lingual epithelium of the dental stalk (Fig. 7A-C). At E13.5, prior to the formation of the second molar, Sox2 was observed in the oral epithelium and at the very distal region making a U-shape surrounding the dental epithelial tip (Fig. 7G). At the same stage Sox9 was widely expressed in the oral and dental epithelium (Fig. 7H). At E13.5, therefore, there is considerable overlap between Sox2 and Sox9 expressing regions. At this stage Sox2^+^ cells were concentrated superficially (Fig. S1A-B), with no labelled cells in the deep planes (Fig. S1C).

### The molar tail tip retains characteristics reminiscent of the initiating molar placode at earlier stages of development

During the initiation of the first molar Sox2 is expressed initially throughout the dental epithelium, then, as the molar epithelium continues to thicken and bud, Sox2 becomes restricted to the lingual side (Juuri et al., 2013b; Zhang et al., 2012). Interestingly, a similar pattern of expression was observed at the posterior tail, with the posterior tip showing widespread Sox2 expression, while the more anterior regions showed a restriction to the lingual side (Fig. 7I,J; level of section in Fig. 7G). At the tip the widespread Sox2 was located in an area of flat epithelium, which thickened anteriorly (Fig. 7I,J). Spatial changes in morphology (thickening to invagination) and changes in Sox2 expression (widespread to lingual) in the epithelial tip of the molar tail from posterior to anterior therefore mimic temporal changes observed during tooth development.

## DISCUSSION

In order to understand how teeth are added sequentially at the back of the mouth it is important to understand the cell dynamics and epithelial topography. Most of the textbook diagrams that describe the morphology of mammalian tooth germs are based on the two-dimensional shape of frontal or sagittal sections. However, the molar dental epithelium morphology is much more complex, as revealed by morphological analysis and 3D reconstructions (this work, Ooe, 1979; Peterkova et al., 2014). Here, we have analysed the posterior epithelial tail of the molar placode, a place where repetitive organ induction occurs to form new molars. We have followed the development and fate of the posterior tail of the first molar (M1) as the second molar (M2) forms. Although our research is confined to the formation of M2 from M1, the conserved morphology of the tail, suggests that a similar set of mechanisms are present in the posterior tail of M2 as the third molar (M3) is formed.

### Fate of the molar tail

We examined the fate of the tail of the first molar by cropping and DiI labelling. We show that the cells at the base of the tail contributed to the formation of the immediate successional molar, with the tip contributing to the third molar. Our results suggest that at E14.5 cells destined to be part of the second and third molar reside in the molar tail. This is in line with the idea that cells for the successional molars are present within the placode in rows at early stages and generate teeth as the tissue grows and elongates. Importantly, new molar development was inhibited after cropping the tail, which suggests a lack of regenerative potential of the remaining tissue to re-form a new tail.

### Oriented cell division, epithelial migration and infolding as possible mechanisms for molar tail growth

As the first molar tail contributes to the formation of new molars we investigated how it elongates posteriorly as the jaw grows. Slightly higher numbers of proliferating cells were detected in the dental epithelial tip compared to the rest of the epithelial tissues of the tail but this was not found to be statistically significant (Fig. 3). It is possible that this subtle difference might reach levels of significance if we increased the number of samples counted. These subtle differences in proliferation rate, however, would appear unlikely to be able to drive the extensive elongation of the molar tail. Changes in proliferation have recently been shown not to underlie other epithelial movements, such as closure of the eyelids during eye development (Heller et al., 2014). Interestingly, epithelial cells can acquire a migratory behaviour during wound healing, and epithelial-mesenchymal transition (Arnoux et al., 2008; Savagner et al., 2005; Thiery et al., 2009). In order to migrate, epithelial cells increase Snail2, reducing cell-cell adhesion by decreasing E-cadherin. A breaking down of the basal lamina occurs in the migratory front. The increased Snail2 and decreased E-cadherin/laminin expression at the molar tail tip therefore suggests a migratory component in the dental epithelium. In view of these results, the tail tip could be considered to be driving the posterior growth of the placode by pushing through the mesenchyme at the back of the jaw. In keeping with this, when the tail tip is cut off, posterior growth is stopped.

Once the tail has elongated, it has to growth mediolaterally. Differences in orientated cell division have been implicated as a mechanism for epithelial morphogenesis in oral epithelium (Economou et al., 2012). Cell division in the tail was shown to be biased in the medio/lateral direction, which coincides with the epithelial cell morphology changes observed in this region. Therefore it is likely that together oriented cell division and delamination contribute to stratification of the dental epithelium of the tail.

It has been described that the posterior aspect of the dental epithelium of a pre-existing molar gives rise to an epithelial bud, which forms the successional molar (Juuri et al., 2013a,b). However, it is not certain from which part of the dental epithelium of the pre-existing molar the successional molar develops. From sagittal views of histological sections, new molars appear to evaginate from the distal part of the outer enamel epithelium (Fig. 7). From our 3D reconstructions this region is also clearly connected to the surface (Fig. 7) and a ribbon of epithelium situated over the tooth germs was observed connected with the posterior bud (Fig. 1F). Our data suggests that this superficial epithelium could be participating in the generation of successional teeth.

Molars can therefore be thought of as having a dual origin, with a contribution from the dental epithelium of the pre-existing tooth (as described by Juuri et al., 2013a,b) but also from the superficial molar tail epithelium. Our clonal analysis using the mT/mG mice, together with the *Snail2*/E-cadherin and laminin expression suggest that a migratory tail is extending backward as the epithelium core incorporates cells by infolding and mitosis. This extension may be particularly important in those unusual mammals that do not restrict themselves to three molars per quadrant but have continuous replacement of teeth posteriorly (Domning and Hayek, 1984; Rodrigues et al., 2011). Although molecular data is not available for these animals, we expect an evolutionary conservation of the expression of migratory markers, such as Snail2, in the posterior dental lamina, maintained throughout the animal's life to allow extension and development of further molars. It would be interesting to assess whether a furrow, as observed here in the mouse, is also evident at the back of the molars in such animals, indicating incorporation of cells from the surface epithelium.

### The molar tail retains characteristics of the early placode

**Fig. 8.**
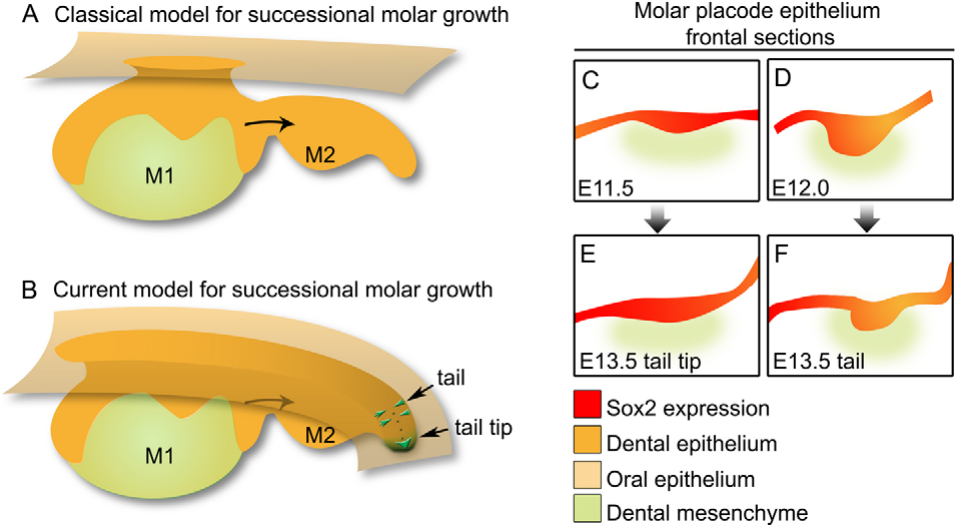
**Model for repetitive tooth formation.** (A) Classical view of a developing molar taken from sagittal histological sections, with the tail projected beneath the oral epithelium. (B) Model proposed by the current work: new teeth are formed from the tail of the molar, which is in continuity with the superficial epithelium. The molar tail is formed by a dual contribution from the dental epithelium associated with the pre-existing molar (Juuri et al., 2013a,b) and the posterior part of the tail. The tail tip moves backward (green tip) as the tail core is refilled by infolding (dotted line) and mediolateral cell protrusion by mitosis (paired arrows). (C-F) Schematic of frontal sections of the most posterior end of the molar tail as indicated in B (based on Fig. 6), in which the epithelial morphology and Sox2 expression is reminiscent of the Sox2 expression at (C) E11.5 (based on Zhang et al., 2012) and (F) E12.0 (based on Juuri et al., 2013b).

The posterior tail of the first molar is comparable to the early initiating molar in morphology: the lingual and labial epithelia of the tail are equivalent to the outer enamel epithelium, and the suprabasal dental epithelium in the tail to the stellate reticulum. Regarding Sox2 expression, spatial changes in the tail tip from posterior to anterior mimic temporal changes as the molar initiates (Figs 7 and 8). Expression of other genes could be studied in the future to confirm whether other dental markers share these temporal and spatial similarities, thereby uniting tooth initiation and successional tooth formation.

The suprabasal epithelium of the tail is in continuity with the stellate reticulum of the tooth germ. The morphology and Sox9 expression pattern of these two tissues suggest that the stellate reticulum derives from the suprabasal epithelium. Interestingly, both the stellate reticulum and the suprabasal epithelium, referred to as the middle epithelium, have been proposed to be a source of stem cells in the mouse incisor (Wang et al., 2007) and in polyphyodont chondrichthyan models (Smith et al., 2009).

### Role of Sox9 and Sox2 in the tail

Similarities between the molar tail and the enamel organ were also observed when Sox2 and Sox9 were compared. Sox2 was mainly localized in the lingual regions of the dental epithelium and Sox9 in the suprabasal dental epithelium and stellate reticulum in the enamel organ of the tooth germ (Fig. 6). At E14.5 Sox2^+^ dental populations were more lingual and continuous with a strongly Sox2^+^ oral epithelium. From E13.5 to E14.5 the expression of Sox2 changed, becoming more restricted to the molar tail and it started to be associated with the deeper planes. As Sox2^+^ cells have been shown to be dental epithelial stem cells (Juuri et al., 2013b), and stem cells derive from other stem cells unless they undergo reprogramming, this change in expression suggests that the progeny of Sox2^+^ cells move to deeper locations. When the Sox9^+^ population was present, Sox2 expression was weak, such as in the labial and suprabasal epithelium and on the aboral side of the lingual dental epithelium. In some organs such as the adult pituitary gland, brain and developing inner ear, Sox2 and Sox9^+^ cells coexist and can represent different progenitor stages (Fauquier et al., 2008; Mak et al., 2009; Poché et al., 2008). Previous research has shown that Sox2^+^/Sox9^−^ cells are stem cell that can derive a Sox2^+^/Sox9^+^ transit-amplifying population representing intermediate progenitors (Fauquier et al., 2008). The same mechanism could be present in the molar tail, as Sox2 and Sox9 are expressed in adjacent domains that overlap slightly. We propose that both Sox2 and Sox9 could participate together in determining the stem/progenitor cell state and control the formation of a new tooth.

## MATERIALS AND METHODS

### Animals and cultures

All experimental procedures were performed following the requirements of the King's College London genetically modified organisms (GMO) committee and the Home Office. Wild type CD-1 mouse embryos were collected from E12.5 to E15.5. For cultures, the mandibular molar region was dissected out and placed on top of transparent nucleopore filters (VWR) supported by metal grids at the surface of Advanced DMEM/F12 culture medium (Invitrogen) supplemented with 1% penicillin/streptomycin, 1% Glutamax (Invitrogen). The explants were incubated at 37°C/5% CO2, and the medium was changed 3 times/week. For the cropping assays, left and right molar regions from the same mandible were dissected with micro surgical blades (WPI) and used as experimental and control treated cultures respectively. For clonal analysis we utilised double-fluorescent Cre reporter mT/mG transgenic mice (Muzumdar et al., 2007). These mice ubiquitously express membrane-targeted tandem Tomato (mT), and after Cre excision express membrane-targeted green fluorescent protein (mG). Pregnant female mice carrying *R26R-mT/mG;R26R-CreER* embryos were injected with 4 mg of tamoxifen at E12.5 and the embryos collected at E14.5. The levels of tamoxifen were adjusted to obtain single cell resolution *in vivo*, generating small clones of mG-positive cells (Muzumdar et al., 2007).

### Fate mapping

For fate mapping, DiI (cell tracker CM-DiI, C-7000, Molecular probes) was re-suspended in 100% ethanol and injected into the tail of the molar placode. The position of the DiI label was followed by stereomicroscope observation during the culture period and in sections after the culture.

### Immunofluorescence and BrdU incorporation

Whole-mount inmunostaining was performed as described (Gaete and Tucker, 2013), with minor modifications: permeabilization and washes were performed using a PBS-Tr solution containing 1% Triton X-100. DNA was stained with DAPI 1:1000 and samples were mounted using Vectashield. The following antibodies and dilutions were used: Sox2 1:300 (2748s, Cell Signalling), Sox9 1:300 (AB 5535, Millipore), Keratin-5 1:500 (PRB-160P-100, Covance), E-cadherin 1:200 (10R-27500, Fitzgerald), Laminin 1:200 (L9393, Sigma). Alexa Fluor 561 and 488 1:500 were used as secondary antibodies. For BrdU assay, intraperitoneal injection of 10 mg/kg of BrdU was performed in a pregnant female carrying E13.5 embryos. The mother was culled and embryos fixed 24 h after injection. Double BrdU and Sox2 detection was performed as described (Gaete et al., 2012) with the same minor modifications described above for immunostaining.

### *In situ* hybridization and immunofluorescence on sections

E14.5 embryos were fixed in 4% PFA at 4°C overnight, washed in PBS/DEPC and dehydrated through a methanol series. For embedding, samples were incubated in isopropanol and cleared in 1,2,3,4 tetrahydronaphtalene then embedded in wax and sectioned at 10 µm. For *in situ* hybridization, sections were rehydrated, refixed in PFA 4% for 20 min, permeabilized in 10 μg/ml Proteinase K for 8 min, acetylated in triethanolamine (T58300, Sigma-Aldrich) plus acetic anhydride (100022M, BDH) for 10 min and dehydrated again prior to the addition of anti-sense probe. The *Snail2* probe (Boutet et al., 2006), kindly provided by Angela Nieto, was used at 1 μg/ml and added in hybridization buffer (50% formamide, 20 mM Tris/DEPC pH 7.5, 300 mM NaCl/DEPC, 5 mM EDTA/DEPC, 1× Denhardt's solution, 10% dextran sulphate, 0.5 mg/ml tRNA) and incubated at 60°C overnight. Samples were washed in 50% formamide–2×SSC, 2×SSC and 0.2×SSC each one twice for 30 min at 60°C. Sections were washed in TN buffer (100 mM Tris pH 7.5, 150 mM NaCl) and blocked in TN buffer with 10% fetal bovine serum (F90665, Sigma) plus 1% BBR (Boehringer, 1096176) for 1 h-RT and incubated with 1:1000 anti-DIG Alkaline Phosphatase antibody (Boehringer, 1093274) overnight at 4°C in blocking solution. Samples were washed in TN buffer for 1 h and incubated in NTMT (100 mM Tris-HCl pH 9.5, 50 mM MgCl2, 100 mM NaCl, 0.1% Tween 20) twice for 10 min. The colour reaction was developed with BM purple (11 442 074 001, Roche).

For immunofluorescence, sections were rehydrated and treated with Tris-EDTA pH 9 for 30 min at 90°C for antigen retrieval. Endogenous peroxidase was quenched with 3% hydrogen peroxide for 30 min. Sections were blocked in TN buffer plus 0.5% BBR, 10% Serum, 1% BSA and 0.05% Tween 20 for 1 h at RT. Anti-Ecad (ab76319, Abcam) was added 1:350 in blocking solution overnight at 4°C. After washing in PBS 0.05% Tween 20, a biotinilated secondary antibody (E0432, Dako) was added at 1:300 for 2 h at RT. A Perkin-Elmer kit (NEL701A001KT) was used for staining according to the manufacturer's instructions and slides were mounted with Fluoroshield DAPI (ab104139, Abcam).

### Image processing quantification and statistics

Slice cultures were photographed during the culture period using a Leica MZ FLIII Stereomicroscope. Samples with immunofluorescence and DiI labelling were scanned on a confocal microscope (Leica SP5 laser scanning confocal microscope). Images were processed using Adobe Photoshop. For cultures smart sharpen filter was applied. Morpho-video was made using the software Sqirlz Morph 2.1 (Xiberpix). Morphing was produced by intercalating software-generated images between original frames to obtain a flowing video. The morphed movies are used for illustrative-academic proposes and do not necessarily represent the exact *ex vivo* development of the molar in culture. Oriented cell division was investigated by detecting matched anaphasic figures in the inverted DAPI staining channel by 3 blind observers (*n*=3). The angle between the axis of anaphases and basal layer was measured using the Fiji angle tool, grouped and plotted using Microsoft Excel. For quantification of BrdU incorporation, optical sections from *z*-stacks were used for each sample. B&C threshold were applied similarly to all samples and epithelial areas were demarcated and counted using the cell counter plug-in in ImageJ (NIH). Results were plotted and analysed by two-way ANOVA and Bonferroni's post-test using Graph Pad Prism 5 software. Fire pseudocolor LUT was used to measure intensity of the signal in ImageJ. 3D views and shadow projections were processed using Imaris ×64 6.3.1 (Bitplane).

## References

[BIO013672C1] Arnoux V., Nassour M., L'Helgoualc'h A., Hipskind R. A. and Savagner P. (2008). Erk5 controls Slug expression and keratinocyte activation during wound healing. *Mol. Biol. Cell* 19, 4738-4749. 10.1091/mbc.E07-10-1078 18716062 PMC2575153

[BIO013672C2] Boutet A., De Frutos C. A., Maxwell P. H., Mayol M. J., Romero J. and Nieto M. A. (2006). Snail activation disrupts tissue homeostasis and induces fibrosis in the adult kidney. *EMBO J.* 25, 5603-5613. 10.1038/sj.emboj.7601421 17093497 PMC1679761

[BIO013672C3] Chlastakova I., Lungova V., Wells K., Tucker A. S., Radlanski R. J., Misek I. and Matalova E. (2011). Morphogenesis and bone integration of the mouse mandibular third molar. *Eur. J. Oral Sci.* 119, 265-274. 10.1111/j.1600-0722.2011.00838.x 21726286

[BIO013672C4] Domning D. P. and Hayek L.-A. C. (1984). Horizontal tooth replacement in the Amazonian manatee (Trichechus inunguis). *Mammalia* 48, 105-128. 10.1515/mamm.1984.48.1.105

[BIO013672C5] Economou A. D., Ohazama A., Porntaveetus T., Sharpe P. T., Kondo S., Basson M. A., Gritli-Linde A., Cobourne M. T. and Green J. B. A. (2012). Periodic stripe formation by a Turing mechanism operating at growth zones in the mammalian palate. *Nat. Genet.* 44, 348-351. 10.1038/ng.1090 22344222 PMC3303118

[BIO013672C6] Economou A. D., Brock L. J., Cobourne M. T. and Green J. B. A. (2013). Whole population cell analysis of a landmark-rich mammalian epithelium reveals multiple elongation mechanisms. *Development* 140, 4740-4750. 10.1242/dev.096545 24173805 PMC3833431

[BIO013672C7] Fauquier T., Rizzoti K., Dattani M., Lovell-Badge R. and Robinson I. C. A. F. (2008). SOX2-expressing progenitor cells generate all of the major cell types in the adult mouse pituitary gland. *Proc. Natl. Acad. Sci. USA* 105, 2907-2912. 10.1073/pnas.0707886105 18287078 PMC2268558

[BIO013672C8] Furuyama K., Kawaguchi Y., Akiyama H., Horiguchi M., Kodama S., Kuhara T., Hosokawa S., Elbahrawy A., Soeda T., Koizumi M. et al. (2011). Continuous cell supply from a Sox9-expressing progenitor zone in adult liver, exocrine pancreas and intestine. *Nat. Genet.* 43, 34-41. 10.1038/ng.722 21113154

[BIO013672C9] Gaete M. and Tucker A. S. (2013). Organized emergence of multiple-generations of teeth in snakes is dysregulated by activation of Wnt/beta-catenin signalling. *PLoS ONE* 8, e74484 10.1371/journal.pone.0074484 24019968 PMC3760860

[BIO013672C10] Gaete M., Muñoz R., Sánchez N., Tampe R., Moreno M., Contreras E. G., Lee-Liu D. and Larraín J. (2012). Spinal cord regeneration in Xenopus tadpoles proceeds through activation of Sox2-positive cells. *Neural Dev.* 7, 13 10.1186/1749-8104-7-13 22537391 PMC3425087

[BIO013672C11] Haraguchi M., Okubo T., Miyashita Y., Miyamoto Y., Hayashi M., Crotti T. N., McHugh K. P. and Ozawa M. (2008). Snail regulates cell-matrix adhesion by regulation of the expression of integrins and basement membrane proteins. *J. Biol. Chem.* 283, 23514-23523. 10.1074/jbc.M801125200 18593711 PMC3259798

[BIO013672C12] Heller E., Kumar K. V., Grill S. W. and Fuchs E. (2014). Forces generated by cell intercalation tow epidermal sheets in mammalian tissue morphogenesis. *Dev. Cell* 28, 617-632. 10.1016/j.devcel.2014.02.011 24697897 PMC4041280

[BIO013672C13] Jernvall J. and Thesleff I. (2012). Tooth shape formation and tooth renewal: evolving with the same signals. *Development* 139, 3487-3497. 10.1242/dev.085084 22949612

[BIO013672C14] Juuri E., Saito K., Ahtiainen L., Seidel K., Tummers M., Hochedlinger K., Klein O. D., Thesleff I. and Michon F. (2012). Sox2+ stem cells contribute to all epithelial lineages of the tooth via Sfrp5+ progenitors. *Dev. Cell* 23, 317-328. 10.1016/j.devcel.2012.05.012 22819339 PMC3690347

[BIO013672C36] Juuri E., Isaksson S., Jussila M., Heikinheimo K. and Thesleff I. (2013a). Expression of the stem cell marker, SOX2, in ameloblastoma and dental epithelium. *Eur. J. Oral Sci.* 121, 509-516. 10.1111/eos.12095 24148099

[BIO013672C15] Juuri E., Jussila M., Seidel K., Holmes S., Wu P., Richman J., Heikinheimo K., Chuong C. M., Arnold K., Hochedlinger K. et al. (2013b). Sox2 marks epithelial competence to generate teeth in mammals and reptiles. *Development* 140, 1424-1432. 10.1242/dev.089599 23462476 PMC3596986

[BIO013672C16] Kavanagh K. D., Evans A. R. and Jernvall J. (2007). Predicting evolutionary patterns of mammalian teeth from development. *Nature* 449, 427-432. 10.1038/nature06153 17898761

[BIO013672C17] Lumsden A. G. (1979). Pattern formation in the molar dentition of the mouse. *J. Biol. Buccale* 7, 77-103. 285076

[BIO013672C18] Mak A. C. Y., Szeto I. Y. Y., Fritzsch B. and Cheah K. S. E. (2009). Differential and overlapping expression pattern of SOX2 and SOX9 in inner ear development. *Gene Expr. Patterns* 9, 444-453. 10.1016/j.gep.2009.04.003 19427409 PMC3023882

[BIO013672C19] Mitsiadis T. A., Mucchielli M.-L., Raffo S., Proust J.-P., Koopman P. and Goridis C. (1998). Expression of the transcription factors Otlx2, Barx1 and Sox9 during mouse odontogenesis. *Eur. J. Oral Sci.* 106 Suppl. 1, 112-116. 10.1111/j.1600-0722.1998.tb02161.x 9541211

[BIO013672C20] Muzumdar M. D., Tasic B., Miyamichi K., Li L. and Luo L. (2007). A global double-fluorescent Cre reporter mouse. *Genesis* 45, 593-605. 10.1002/dvg.20335 17868096

[BIO013672C21] Nanci A. (2008). *Ten Cate's Oral Histology: Development, Structure, and Function*. Mosby Inc, an affiliate of Elsevier Inc, China.

[BIO013672C22] Ooe T. (1979). Development of human first and second permanent molar, with special reference to the distal portion of the dental lamina. *Anat. Embryol.* 155, 221-240. 10.1007/BF00305754 420409

[BIO013672C23] Panousopoulou E. and Green J. B. A. (2014). Spindle orientation processes in epithelial growth and organisation. *Semin. Cell. Dev. Biol.* 34, 124-132. 10.1016/j.semcdb.2014.06.013 24997348

[BIO013672C24] Peterkova R., Hovorakova M., Peterka M. and Lesot H. (2014). Three-dimensional analysis of the early development of the dentition. *Aust. Dent. J.* 59 Suppl. 1, 55-80. 10.1111/adj.12130 24495023 PMC4199315

[BIO013672C25] Poché R. A., Furuta Y., Chaboissier M.-C., Schedl A. and Behringer R. R. (2008). Sox9 is expressed in mouse multipotent retinal progenitor cells and functions in Müller glial cell development. *J. Comp. Neurol.* 510, 237-250. 10.1002/cne.21746 18626943 PMC4412477

[BIO013672C26] Prochazka J., Pantalacci S., Churava S., Rothova M., Lambert A., Lesot H., Klein O., Peterka M., Laudet V. and Peterkova R. (2010). Patterning by heritage in mouse molar row development. *Proc. Natl. Acad. Sci. USA* 107, 15497-15502. 10.1073/pnas.1002784107 20709958 PMC2932592

[BIO013672C27] Rizzoti K., Akiyama H. and Lovell-Badge R. (2013). Mobilized adult pituitary stem cells contribute to endocrine regeneration in response to physiological demand. *Cell Stem Cell* 13, 419-432. 10.1016/j.stem.2013.07.006 24094323 PMC3793864

[BIO013672C28] Rodrigues H. G., Marangoni P., Sumbera R., Tafforeau P., Wendelen W. and Viriot L. (2011). Continuous dental replacement in a hyper-chisel tooth digging rodent. *Proc. Natl. Acad. Sci. USA* 108, 17355-17359. 10.1073/pnas.1109615108 21987823 PMC3198356

[BIO013672C29] Savagner P., Kusewitt D. F., Carver E. A., Magnino F., Choi C., Gridley T. and Hudson L. G. (2005). Developmental transcription factor slug is required for effective re-epithelialization by adult keratinocytes. *J. Cell. Physiol.* 202, 858-866. 10.1002/jcp.20188 15389643

[BIO013672C30] Scott C. E., Wynn S. L., Sesay A., Cruz C., Cheung M., Gomez Gaviro M.-V., Booth S., Gao B., Cheah K. S. E., Lovell-Badge R. et al. (2010). SOX9 induces and maintains neural stem cells. *Nat. Neurosci.* 13, 1181-1189. 10.1038/nn.2646 20871603

[BIO013672C31] Sequeira I. and Nicolas J.-F. (2012). Redefining the structure of the hair follicle by 3D clonal analysis. *Development* 139, 3741-3751. 10.1242/dev.081091 22991440

[BIO013672C32] Smith M. M., Fraser G. J. and Mitsiadis T. A. (2009). Dental lamina as source of odontogenic stem cells: evolutionary origins and developmental control of tooth generation in gnathostomes. *J. Exp. Zool. B Mol. Dev. Evol.* 312B, 260-280. 10.1002/jez.b.21272 19156674

[BIO013672C33] Thiery J. P., Acloque H., Huang R. Y. J. and Nieto M. A. (2009). Epithelial-mesenchymal transitions in development and disease. *Cell* 139, 871-890. 10.1016/j.cell.2009.11.007 19945376

[BIO013672C34] Wang X.-P., Suomalainen M., Felszeghy S., Zelarayan L. C., Alonso M. T., Plikus M. V., Maas R. L., Chuong C.-M., Schimmang T. and Thesleff I. (2007). An integrated gene regulatory network controls stem cell proliferation in teeth. *PLoS Biol.* 5, e159 10.1371/journal.pbio.0050159 17564495 PMC1885832

[BIO013672C35] Zhang L., Yuan G., Liu H., Lin H., Wan C. and Chen Z. (2012). Expression pattern of Sox2 during mouse tooth development. *Gene Expr. Patterns* 12, 273-281. 10.1016/j.gep.2012.07.001 22835638

